# Integrating clinical and multiomics evidence based on disease module theory: deciphering the comorbidity network of psoriasis vulgaris via the Ising model for mechanistic insights

**DOI:** 10.3389/fimmu.2026.1744789

**Published:** 2026-04-14

**Authors:** Dongwen Shi, Yuan Luo, Ping Zhang, Bin Liang, Mengmeng Wang, Hong Cai, Xiaowen Pang

**Affiliations:** 1 Department of Dermatology, Air Force Medical Center, Air Force Medical University, PLA, Beijing, China; 2 Laboratory of Clinical Medicine, Air Force Medical Center, Air Force Medical University, PLA, Beijing, China

**Keywords:** comorbidity network, disease module, immune-mediated inflammation, ising model, multiomics analysis, psoriasis vulgaris

## Abstract

Psoriasis vulgaris (PV), a chronic immune-mediated inflammatory dermatosis, is associated with a significant burden of systemic comorbidities. Traditional comorbidity research methods struggle to reveal its complex interconnectedness. Based on large-scale retrospective cohort data, we constructed a PV comorbidity network using the Ising model from statistical physics. Weighted network centrality analysis was used to identify core and hub nodes and elucidate shared molecular mechanisms at the multiomics level (nontargeted proteomics and lipid peroxidation metabolomics). Finally, the impact of IL-17A inhibition (IL-17Ai) on PV and atherosclerosis (assessed by carotid Doppler color ultrasound) was evaluated using a prospective intervention study. The Ising model identified atherosclerosis- coronary heart disease (CHD) as the core comorbidity (degree centrality >10), with pulmonary nodules, hypertension, and fatty liver serving as key hub nodes (betweenness centrality >60). Multiomics analysis revealed a core molecular mechanism in PV, involving immune inflammation, oxidative stress, lipid metabolism disorder, and coagulation abnormalities, where the oxidative stress molecule GPX3 acts as a critical hub. Following IL-17Ai intervention, both skin lesions and early atherosclerosis markers significantly improved, accompanied by downregulation of the proinflammatory peripheral blood factor S100A9 and upregulation of anti-inflammatory lipid peroxidation metabolites (e.g., 17(R)-RVD1). This study systematically revealed the modular hierarchical structure of PV comorbidities at the network topology and molecular mechanism levels, confirming the central role of the IL-17 signaling pathway in driving the comorbidity network. This conclusion was further clinically validated by IL-17Ai intervention outcomes. This research provides theoretical and clinical evidence for early identification, prioritized management, and "one drug, multiple targets" therapeutic strategies for treating PV comorbidities.

## Graphical Abstract

**Figure:**
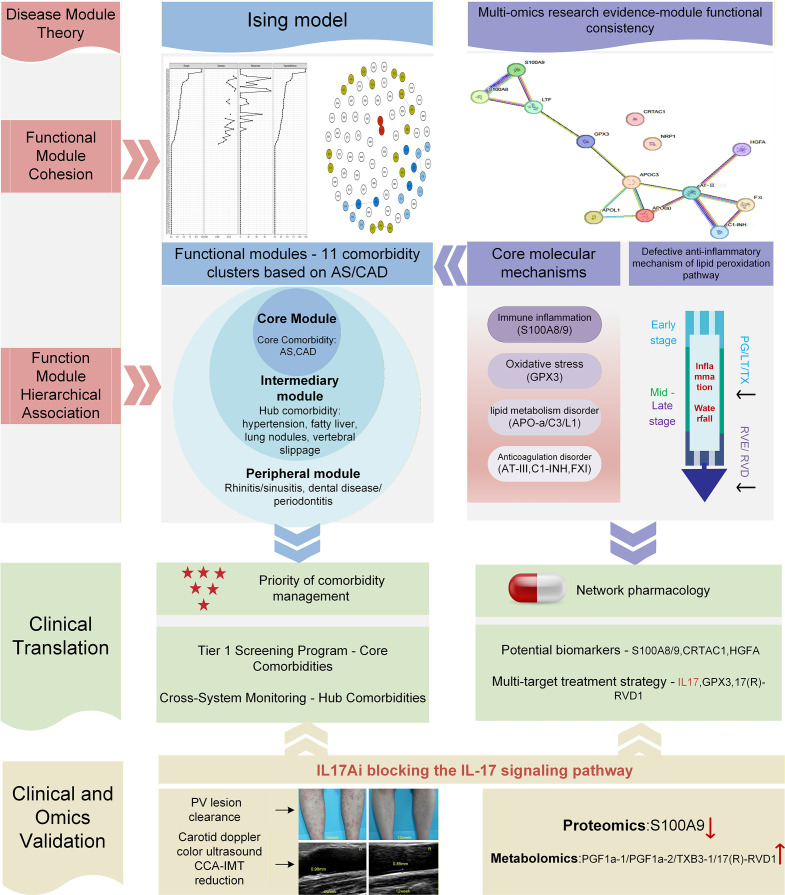

## Highlights

- For the first time, the statistical mechanics Ising model was applied to PV comorbidity network analysis. By using “coupling strength (Jij)” to replace traditional frequency-based approaches, it precisely quantifies functional dependencies between comorbidities; identifies core comorbidities (atherosclerosis, coronary artery disease) and hub comorbidities (pulmonary nodules, hypertension, fatty liver, etc.) in PV; addresses the key challenge of “difficulty in quantifying comorbidity interaction effects”; and provides a methodological reference for comorbidity studies in other complex diseases (such as rheumatoid arthritis and diabetes).
- Established a comprehensive research paradigm spanning “disease module theory — Ising model construction (using large-sample clinical data) — carotid ultrasound validation (clinical phenotypes) — nontargeted proteomics/lipid peroxidation metabolomics analysis (molecular mechanisms).” This achieves systematic integration from the “macro comorbidity network—micromolecular mechanisms—clinical intervention effects.”
- Advanced the application of “disease module theory” in dermatology: First, a hierarchical structure was demonstrated in the PV comorbidity network, comprising “core module (atherosclerosis, coronary artery disease) → hub module (pulmonary nodules, hypertension, etc.) → peripheral module (gastritis, rhinitis, dental diseases, etc.).” These findings reveal that PV is fundamentally driven by “systemic inflammation-promoting cross-system module associations,” offering a new theoretical framework for understanding comorbidity mechanisms in complex diseases.
- Defined “management priorities” for PV comorbidities (with core comorbidities as primary screening targets and hub comorbidities as key focuses for cross-system monitoring). Recommended regular monitoring, such as coronary CT, carotid ultrasound, and lung CT, directly equips clinicians with practical diagnosis and treatment strategies.
- Potential biomarkers such as S100A8/A9 (reflecting PV inflammatory activity), CRTAC1 (associated with bone and joint comorbidities), and HGFA (linked to fatty liver) were identified. Proposed therapeutic targets, such as the IL-17 pathway, GPX3 (an oxidative stress hub), and 17(R)-RVD1 (a lipid peroxidation anti-inflammatory factor), lay the groundwork for the precise diagnosis of PV and its comorbidities and support “one-drug multitarget” treatment approaches.

## 1 Introduction

Psoriasis vulgaris (PV) is a chronic, immune-mediated inflammatory skin disease with a global prevalence of approximately 2–3% (1). Beyond its cutaneous manifestations, PV is increasingly recognized as a systemic disorder characterized by a significant burden of comorbidities affecting multiple systems, including cardiovascular (e.g., atherosclerosis, coronary heart disease, hypertension) (2), metabolic (e.g., diabetes, hyperlipidemia, hyperuricemia) (3), digestive, respiratory, and musculoskeletal systems (4, 5). This intricate interplay suggests underlying shared pathophysiological mechanisms rather than mere coincidence.

Traditional comorbidity research methods—such as prevalence statistics, logistic regression, and co-occurrence analysis—primarily quantify pairwise associations. While valuable, these approaches struggle to capture the intrinsic interconnectedness among multiple comorbidities and fail to model the dynamic interactions and synergistic effects present in complex networks. The “disease modules” theory proposed by Barabási et al. offers a new framework for understanding this complexity (6). This theory posits that complex diseases are not isolated abnormalities of single molecules or clinical phenotypes but rather “modules” formed by a group of highly functionally connected nodes (such as genes, proteins, or diseases) within biological networks. The essence of comorbidity lies in the interconnection of different disease modules through key “hub nodes,” forming a cross-system associative network. This provides a powerful theoretical model for understanding the systemic inflammatory nature of PV (7) and its multisystem comorbidity. However, the underlying mechanisms linking PV to cardiovascular, metabolic, musculoskeletal, and other multisystem comorbidities remain poorly understood at the network level.

To address this limitation, this study pioneers the application of the Ising model from statistical physics as a novel tool for quantifying PV comorbidity associations. Successfully applied by Wang et al. in 2022 for module detection in complex diseases (8), this model’s core advantage lies in transcending traditional co-occurrence frequency. It quantifies functional dependencies between comorbid nodes through “coupling strength” and combines weighted network centrality analysis (e.g., degree centrality and betweenness centrality) to intuitively and precisely identify core driver nodes and cross-module interaction hubs within the network. This provides a robust “mechanism modeling paradigm” for constructing and quantifying a PV comorbidity network topology at the macro level.

At the micromechanism level, proteomics and lipid peroxidation metabolomics serve as ideal tools for exploring the complex biological systems of PV (9). Nontargeted proteomics can unbiasedly reveal differentially expressed proteins and key pathways between PV patients and healthy individuals (10), whereas lipid peroxidation metabolomics directly targets oxidative stress—a critical pathway in PV pathogenesis (11). Deciphering changes in relevant metabolites provides crucial evidence for elucidating the shared molecular mechanisms of comorbidities. Integrating multiomics data holds promise for offering valuable biological interpretations of the network associations revealed by the Ising model.

More importantly, at the clinical practice level, previous studies have clearly established a strong association between atherosclerotic cardiovascular disease and psoriasis (PV), posing serious health and life threats to affected patients (12). Clinical guidelines co-issued by the American Academy of Dermatology (JAAD) and the National Psoriasis Foundation (NPF) explicitly emphasize that individuals with psoriasis face a significantly elevated risk of developing cardiovascular disease. Notably, atherosclerosis is not merely a passive metabolic disorder but an active inflammatory process. The core mechanism linking psoriasis and atherosclerosis involves the shared participation and regulation of immune-inflammatory cells and associated cytokines (13). The hypothesis that psoriasis-activated inflammatory cascades drive the progression of atherosclerosis provides a theoretical rationale for improving atherosclerotic outcomes and reducing the risk of major adverse cardiovascular events (MACEs) through anti-inflammatory therapy (14). However, the specific effects of interleukin-17A inhibitors (IL-17Ai)—a key targeted anti-inflammatory treatment for psoriasis—on atherosclerosis remain controversial (15). Currently, clinical practice lacks both reliable biomarkers that clearly indicate cardiovascular risk progression and tailored cardiovascular risk assessment guidelines or models for the psoriatic population. This gap significantly hinders the early identification and timely intervention of cardiovascular risk factors in these patients. Altogether, this situation highlights an urgent clinical need to explore, develop, and evaluate treatment strategies that effectively address both psoriasis and its associated cardiovascular comorbidities.

Therefore, this study employs an integrated macromicro strategy: First, leveraging large retrospective cohort data, an Ising model is used to construct a PV comorbidity network to visualize and quantify its topological structure and identify key core and hub comorbidities; subsequently, a prospective intervention study targeting PV and its most clinically significant cardiovascular comorbidities is conducted. In this study, clinical imaging (carotid ultrasound) is combined with multiomics technologies (nontargeted proteomics and lipid peroxidation metabolomics) to dynamically assess the impact of IL-17Ai intervention on PV and atherosclerosis within a longitudinal framework. This approach involves a preliminary exploration of the core molecular mechanisms underlying the interactions and effects among PV, its comorbidities, and targeted drug therapies. This research provides not only novel network theory and molecular mechanism perspectives for understanding the systemic disease nature of PV but also potential experimental evidence and theoretical support for early comorbidity identification, prioritization of clinical interventions, and novel drug development.

## 2 Materials and methods

### 2.1 Study population

#### 2.1.1 Data source, study population, and subjects for retrospective cohort study

**Figure 1:**
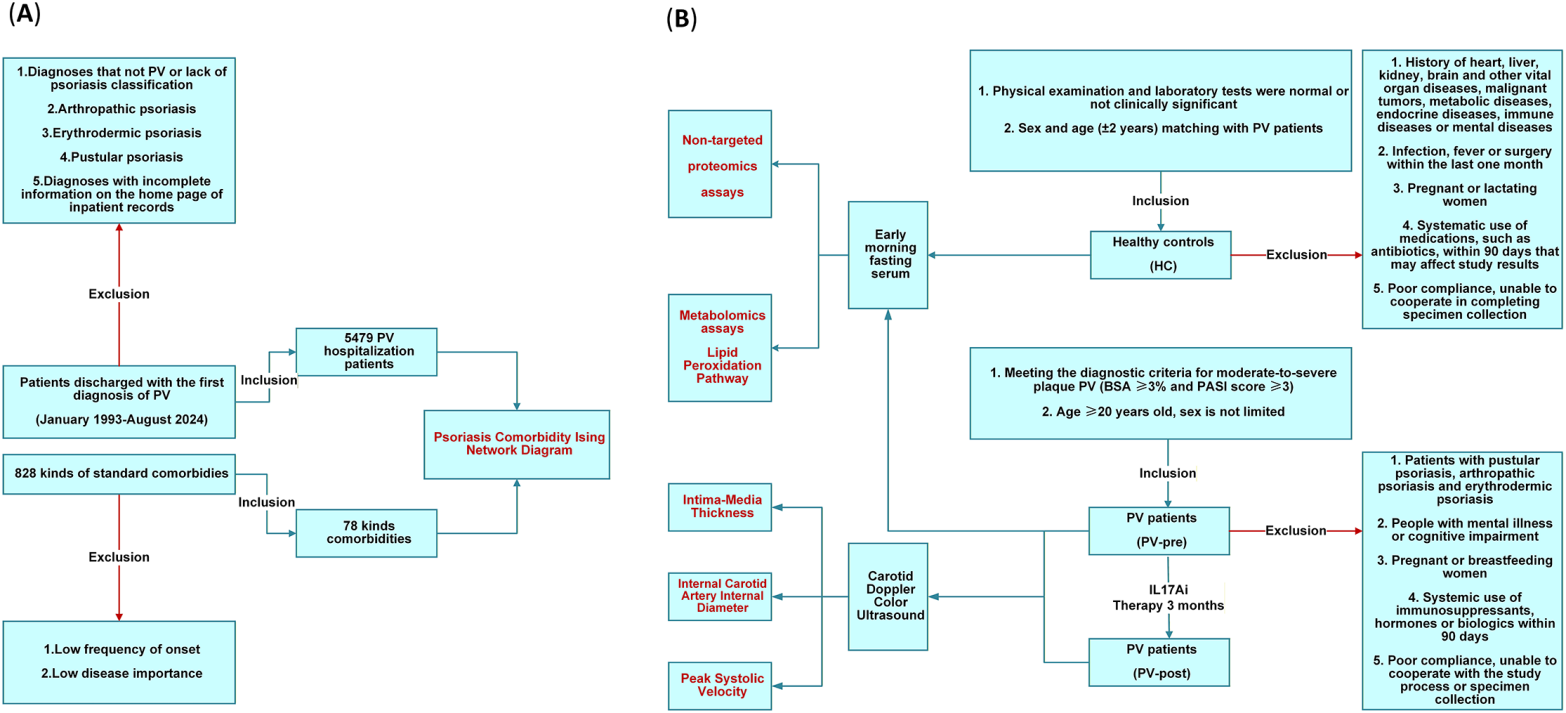
Flowchart.

Data for constructing the PV comorbidity network Ising model were sourced from the Hospital Information System (HIS) of the Air Force Medical Center of the People’s Liberation Army. This system comprehensively records demographic information, clinical diagnosis and treatment details, and nursing care records for all inpatients. Medical record quality is supervised and reviewed by the National Healthcare Security Administration and the hospital’s Medical Record Quality Management Committee. All the research data were anonymized and deidentified; patient privacy information in compliance with the Personal Information Protection Law of the People’s Republic of China was removed, and human subjects were not included. The study population comprised 5,479 hospitalized patients (**Supplementary Table 1**) discharged between January 4, 1993, and August 17, 2024, with PV as the primary diagnosis on the discharge summary, selected according to the inclusion and exclusion criteria. Referencing the International Classification of Diseases (ICD) 10 coding system, 78 comorbidities across 19 major categories were selected for study on the basis of their frequency of occurrence in PV, findings from prior PV comorbidity studies, and disease significance. Low-frequency and low-clinical-significance comorbidities were excluded (**Figure 1**; **Supplementary Table 2**; **Supplementary Materials #10- Sheet1**, **2**).

#### 2.1.2 Study population and sample collection for the prospective intervention cohort

In accordance with the inclusion and exclusion criteria (**Figure 1A**), 30 patients with moderate-to-severe plaque-type PV who visited the dermatology outpatient clinic at the Air Force Medical Center between January 1, 2024, and September 1, 2024, were selected. Participants received subcutaneous injections of 300 mg of the IL-17 antagonist secukinumab (Novartis Pharmaceuticals, USA) at 0, 1, 2, 3, 4, 8, and 12 weeks. Concurrently, they received symptomatic treatment with oral antihistamines for pruritus and topical emollients. 12 weeks. Concurrently, symptomatic treatment included oral antihistamines for pruritus and topical emollients (boric acid ointment, moisturizing cream, urea cream, petroleum jelly, etc., prepared by the Air Force Medical Center Compounding Laboratory, China). Quality of life and disease severity assessments (DLQI, BSA, and PASI) were conducted at weeks 0 and 12. Safety monitoring included complete blood count, urinalysis, liver function tests, renal function tests, lipid profiles, 8-item surgical infection panels, comprehensive tumor marker panels, full-length IGRAs, ANAs, and lung CT scans), and carotid Doppler color ultrasound (Philips EPIQ7, probe model L12-5, frequency 5–12 MHz). Ultrasound parameters included the common carotid artery intima-media thickness (CCA-IMT), internal carotid artery lumen diameter (ICA-LD), and peak systolic velocity (PSV) (**Supplementary Table 3**). Patients also received health education and management, including dietary guidance, sleep management, smoking cessation, alcohol avoidance, and aerobic exercise. Twenty-one patients completed the 12-week clinical observation and assessment protocol.

Among 21 patients with moderate-to-severe PV, 10 (enrolled between March 6, 2024, and August 27, 2024) underwent pre- and posttreatment multiomics testing. Concurrently, 10 healthy controls matched for sex and age (± 3 years) to PV patients were selected from our center’s health examination center. All participants were informed of the study objectives and provided written informed consent. This study was approved by the Clinical Ethics Committee of the Air Force Medical Center (Approval No.: 2023-93-PJ01) and complies with the principles of the Declaration of Helsinki. Blood samples were collected from the healthy control (HC), PV pretreatment (PV-pre), and PV posttreatment (PV-post) groups after an 8-hour overnight fast. Five milliliters of venous blood was drawn into vacuum anticoagulant tubes and allowed to stand at room temperature (22–25 °C) for 30–60 minutes. Following centrifugation, 300 μL of the serum supernatant was collected and stored at -80 °C (**Figure 1B**).

### 2.2 Construction of the Ising model for PV cohort comorbidities

This study is a single-center, retrospective cohort study. After screening 5,479 PV patients and their 78 comorbidities on the basis of the inclusion and exclusion criteria, rigorous quality control and data cleaning were performed on the collected clinical data. Each comorbidity was treated as a binary variable (present = 1, absent = 0), forming a 78×5479 comorbidity-patient matrix. Nodes represent the 78 comorbidities, whereas edges indicate dependency relationships between comorbidities (analogous to “spin particle interactions” in statistical mechanics). The binary Ising fit function from statistical mechanics was applied to calculate the coupling strength (J_(ij)) on the basis of the “spin coupling” of PV comorbidity co-occurrence. Since cross-sectional data eliminate the need to simulate dynamic evolution, the temperature (T) and external magnetic field (H) from the physical model were omitted, with a focus purely on the correlations between comorbidities. The coupling strength function is simplified to J_(ij) = Log(P(i)P(j)P(i∩j)), where P(i∩j) is the probability of comorbidities i and j occurring simultaneously and P(i) and P(j) are their individual occurrence probabilities (corresponding to the log-likelihood ratio of “spin cooperative orientation” in statistical mechanics). Using maximum likelihood estimation, we quantify the “nonrandom co-occurrence probability” of comorbidities i and j. A larger absolute value of the coupling strength indicates a stronger tendency for comorbidities i and j to occur synergistically (16).

### 2.3 Nontargeted proteomics and lipid peroxidation metabolomics experimental methods, reagents, and instruments

This study is a prospective intervention cohort study. Peripheral serum multiomics testing for healthy controls and PV patients before and after treatment followed internationally standardized protocols (17–19). Specific methods, reagents, and instruments are detailed in the supplementary materials (**Supplementary Materials -#11** Supplementary Methods; **Supplementary Table 4**).

### 2.4 Statistical analysis

Normally distributed quantitative data are expressed as the mean ± standard deviation. Nonnormally distributed quantitative data are presented as medians (interquartile ranges). Count data are reported as percentages. Paired or grouped t tests/rank-sum tests were used to compare quantitative data, including age, body mass index (BMI), DLQI, PASI, BSA, carotid ultrasound parameters, and quantitative results of peripheral blood differential proteins and metabolites. Two-tailed tests were performed, with P < 0.05 indicating a statistically significant difference.

PV-weighted comorbidity network topology analysis using the Ising model: The Ising model was transformed into a weighted network where edge weights corresponded to coupling strengths Jij. Node centrality (Centrality) was assessed and analyzed on the basis of weighted centrality metrics derived from Jij. Degree centrality (strength) represents the total coupling strength of a node (comorbidity), reflecting its influence within the network; expected influence measures a node’s importance in connecting to “high-coupling-strength nodes,” reflecting its global influence and diffusion potential. Betweenness centrality quantifies how frequently a node acts as a ‘bridge’ connecting different comorbidities, indicating its intermediary role in “high-coupling-strength paths.” Closeness centrality: The average coupling strength distance from a node to all other nodes reflects its information dissemination efficiency. Clustering was performed using a coupling strength threshold (J > 0.5) (**Supplementary Materials-#10 Sheet 19**) to delineate distinct comorbidity clusters (disease modules). The original cohort was divided into two subsets based on discharge time: “1993–2014” and “2015–2024.” Ising models were constructed for each subset (**eFigure 1**). Compare the fundamental characteristics of two temporal subset queues and analyze the sample sizes and network correlations (edge weight correlations, node strength centrality correlations, etc.) between the two subsets to assess model stability.

Definition and screening of differentially expressed proteins and metabolites: Fold change (FC) values were calculated using fold change analysis, defined as the ratio of PV-post/PV-pre or PV-post/PV-pre for protein and metabolite levels, respectively. Genes with FC values ≥1.20 or ≤0.86 were defined as differentially expressed proteins or metabolites. Orthogonal partial least squares discriminant analysis (OPLS-DA) was employed for classification. Model reliability was validated through cross-validation and permutation testing. Differential proteins and metabolites were screened on the basis of the P value (P < 0.05) and variable importance in projection (VIP > 1). Multiple testing correction using the Benjamini-Hochberg method (False Discovery Rate, FDR) was applied to all differentially expressed molecules (**Supplementary Materials - #12** Supplementary Methods).

Protein interaction analysis of the selected differentially expressed proteins was performed using the STRING database (https://cn.string-db.org/). Functional classification, cellular localization, and pathway enrichment analyses of the selected differentially expressed proteins were conducted using the Gene Ontology (GO) and Kyoto Encyclopedia of Genes and Genomes (KEGG) databases.

All the statistical analyses in this study were performed using SPSS (version 27.0) and R (version 4.3.2).

## 3 Results

### 3.1 Topological analysis of PV cohort comorbidity networks based on the Ising model

#### 3.1.1 General characteristics of the PV patient cohort

A total of 5,479 PV patients were included, with discharge dates ranging from January 4, 1993, to August 17, 2024 (for PV patients hospitalized ≥2 times, data from the first hospitalization were used). The hospitalization duration ranged from 1 to 106 days, with a mean of 16.6 ± 10.6 days. There were 3,990 male patients and 1,489 female patients. The ages ranged from 2 to 89 years, with a mean age of 38.5 ± 16.4 years.

#### 3.1.2 Topological features of the comorbidity network in the PV cohort

**Figure 2:**
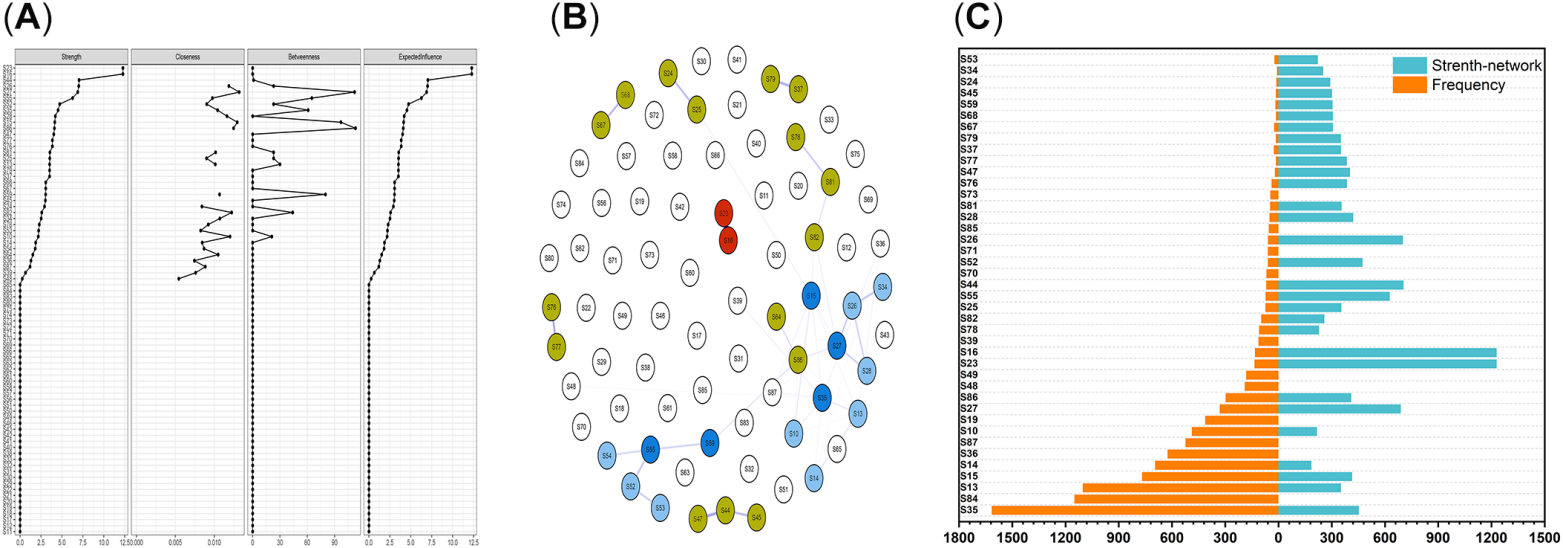
Topology structure and frequency analysis of PV comorbidity based on the Ising model. **(A)** Centrality ranking of the PV comorbidity weighted network based on the Ising model, where Strength denotes degree centrality, Betweenness denotes betweenness centrality, Expected Influence denotes expected influence centrality, and Closeness denotes closeness centrality. **(B)** Topological structure diagram of the PV comorbidity coupling network transformed from the Ising model, with nodes represented by circles and each node corresponding to a comorbidity name; lines between circles serve as bridges, with line thickness indicating coupling strength Jij magnitude. Jij > 0.5 denotes a strong association (**Supplementary Materials-#10 Data-sheet 19**), resulting in the formation of 11 comorbidity clusters. Core comorbidity clusters (red, degree centrality > 10): Atherosclerosis (S23)-Coronary Heart Disease (S16); Hub comorbidity cluster (blue; 5 hub comorbidities with betweenness centrality >60: hypertension, fatty liver, pulmonary nodules, osteoporosis, and spondylolisthesis, shown in dark blue): Hypertension (S15) - Fatty Liver (S35) - Type 2 Diabetes (S10) - Hyperlipidemia (S13) - Hyperuricemia (S14), pulmonary nodules (S27)-emphysema (S26)-bullous emphysema (S28)-bronchiectasis (S34), osteoporosis (S59)-spondylolisthesis (S55)-cervical spondylosis (S54)-degenerative osteoarthropathy (S52)-fasciitis/tenosynovitis/synovitis (S53); Peripheral Comorbidity Cluster (Turmeric Yellow): Arterial plaque (S24) - Stroke (S25), Gastritis (S44) - Peptic ulcer (S45) - Intestinal polyps (S47), Liver cirrhosis (S37) - Thrombocytopenia (S79), Anemia (S78) - Hypoproteinemia (S81) - Electrolyte Disturbance (S82), Thyroid Dysfunction (S64) - Benign Tumor (S86), Dental Disease (S76) - Periodontitis (S77), Rhinitis (S67) - Sinusitis (S68). **(C)** Cumulative frequency histogram of PV comorbidities and Ising model comorbidity network strength. Vertical axis: 41 comorbidities derived from the intersection of the top 30 comorbidities by frequency (**Supplementary Table 2**) and the top 30 by Ising network model strength **(A)**. Horizontal axis: The left side shows the comorbidity frequency, and the right side shows the network strength (×100).

A coupled topological network derived from the Ising model was constructed for 78 comorbidities within the 5,479-patient PV cohort (**Figure 2**) This network revealed the following:

Strength centrality, which serves as an indicator of comorbidity intensity within the network, differs from comorbidity frequency rankings (**Figure 2C**). The top 10 PV comorbidities ranked by influence strength were atherosclerosis (12.3), coronary heart disease (12.3), gastritis (7.0), emphysema (7.0), pulmonary nodules (6.9), spondylolisthesis (6.3), degenerative osteoarthritis (4.7), fatty liver (4.5), bullous emphysema (4.2), and hypertension (4.1). Atherosclerosis and coronary heart disease are at the top two positions and are strongly associated with mutual comorbidity. Both conditions demonstrate degree centrality values exceeding 10, establishing them as the most critical core comorbidities of PV. The expected influence centrality results strongly align with the degree centrality findings, indicating that the “local activity” and “global hub status” of core comorbidities coincide. These findings further validate the central position of atherosclerosis and coronary heart disease within the comorbidity network.

Betweenness centrality reflects the mediating and bridging roles of comorbidities within the network system. Comorbidities with values exceeding 60 were benign tumors, pulmonary nodules, hypertension, osteoporosis, spondylolisthesis, and fatty liver disease. In addition to benign tumors (a nonsingle disease), pulmonary nodules, hypertension, fatty liver disease, spondylolisthesis, and osteoporosis emerged as pivotal hubs connecting comorbidities. Comorbidities with closeness-to-center scores exceeding 0.0125—primarily pulmonary nodules and hypertension—demonstrated high information transmission efficiency within the network. With pulmonary nodules as the central node, four closely associated comorbidity clusters emerge: Emphysema-Bullous Emphysema-Bronchiectasis - Fatty Liver-Hyperlipidemia-Hyperuricemia-Type 2 Diabetes-Hypertension - Electrolyte Imbalance-Hypoproteinemia-Anemia - Thyroid Dysfunction-Benign Tumors Centered on hypertension; the associated comorbidity clusters and conditions include type 2 diabetes-fatty liver-hyperlipidemia-hyperuricemia, stroke-arterial plaque, electrolyte imbalance-hypoproteinemia-anemia, and cysts. Fatty liver disease occupies a pivotal position only within the hypertension-type 2 diabetes-hyperlipidemia-hyperuricemia (metabolic syndrome) comorbidity cluster. Additionally, closely interconnected comorbidity clusters in the PV cohort included osteoporosis-spondylolisthesis-cervical spondylosis-degenerative osteoarthritis-ligament tendon sheath fasciitis synovitis, gastritis-intestinal polyps-peptic ulcer, cirrhosis-thrombocytopenia, dental disease-periodontitis, and rhinitis-sinusitis.

#### 3.1.3 Data subset validation

The original PV cohort (5,479 cases) was divided into two subsets on the basis of discharge dates: “1993–2014” and “2015–2024.” Comparing sample size, age, sex, total comorbidities, and the number of top 10 comorbidities between the two temporal subset cohorts revealed no statistically significant differences in sex and the prevalence of comorbidities such as type 2 diabetes, hyperlipidemia, hypertension, arrhythmia, abnormal liver function, and cysts. Although statistically significant differences were observed in age, hyperuricemia, fatty liver, and infectious diseases, the effect sizes were small. This suggests these differences likely stem primarily from the large sample size rather than representing clinically meaningful substantive changes. Notably, the difference in the proportion of pulmonary nodules between the two groups was statistically significant with a moderate effect size. This may be related to advances in imaging detection technology (the application of high-resolution CT in later periods improved detection rates) and the COVID-19 pandemic beginning in 2019, which significantly increased public awareness of pulmonary lesions (**Supplementary Table 5**). Ising networks were constructed for each subset, revealing greater than 85% consistency in centrality rankings for core comorbidities across both subsets. Network correlation analysis between the two subsets revealed the following: edge weight correlation coefficient r = 0.7642, P < 0.0001, 95% CI: 0.7535 to 0.7745, indicating highly correlated edge weight patterns across both periods; the correlation coefficient for node strength centrality was r = 0.7179, P < 0.0001, with a 95% confidence interval of 0.5896 to 0.8109, indicating that the importance rankings of nodes in the networks across the two periods maintained good stability (**eFigure 1**; **Supplementary Materials- Sheet 3**, **4**).

### 3.2 Clinical efficacy and carotid doppler color ultrasound analysis

We evaluated the clinical efficacy, safety, and carotid Doppler color ultrasound findings in 21 patients with moderate-to-severe plaque-type PV before and after 12 weeks of standardized IL-17Ai therapy. The cohort included 21 PV patients (14 males, 7 females) ranging from 23.0 to 70.0 years old, with a mean age of 43.1 ± 15.7 years; the baseline body mass index (BMI) ranged from 19.9 to 38.8 kg/m², with a mean of 25.9 ± 4.1 kg/m²; 10 patients (47.6%) had a history of smoking; and 4 patients (23.8%) had a history of alcohol consumption; 13 cases (61.9%) of insufficient sleep. Ultrasound revealed carotid intima-media thickening/plaques/lumen stenosis in 9 patients (42.9%). After 12 weeks of IL-17Ai treatment, all 21 PV patients demonstrated significant reductions in quality of life and disease severity indices (DLQI, BSA, and PASI) (P < 0.001) (**Supplementary Table 6**), with no notable adverse reactions observed. Doppler color ultrasound revealed significant improvement from baseline values after treatment, when adjusted for lifestyle modifications (smoking, alcohol consumption, and sleep deprivation) as covariates: reduced common carotid artery intima-media thickness (IMT, P < 0.05), increased internal carotid artery lumen diameter (ID-ICA, P < 0.05), and a trend toward decreased carotid systolic peak velocity (PSV) (**Table 1**).

**Table 1:** Comparison of color Doppler ultrasound parameters of the carotid artery before and after IL-17Ai treatment in PV patients.

Ultrasonic parameters of the carotid artery		PV-pre (n=21)	PV-post (n=21)	Adjusted coefficient (B)	*p*-value	95% confidence interval
		x̄ ± s	x̄ ± s			
CCA-IMT (mm)	left	0.917 ± 0.213	0.868 ± 0.217	-0.058	0.014	-0.102, -0.013
	right	0.951 ± 0.202	0.879 ± 0.213	-0.067	0.033	-0.128, -0.006
ICA-LD (mm)	left	4.857 ± 0.489	4.967 ± 0.394	0.063	0.676	-0.248, 0.373
	right	4.857 ± 0.418	5.091 ± 0.379	0.262	0.027	0.033, 0.492
PSV (cm/s)	left	80.857 ± 23.359	74.429 ± 15.642	-4	0.552	-17.910, 9.910
	right	76.667 ± 23.150	75.238 ± 13.420	-1.31	0.818	-13.180, 10.550

PV, psoriasis vulgaris. PV-pre, Pre-IL-17Ai treatment group; PV-post, Post-IL-17Ai treatment group. $\bar{x}$, mean; *s*, standard deviation. CCA-IMT, common carotid artery intima–media thickness; ICA-LD, internal carotid artery lumen diameter; PSV, peak systolic velocity. Adjusted Coefficient (B) was derived from linear regression models, adjusting for Smoke, Drink and Sleep. Data are presented as Mean ± Standard Deviation for unadjusted change, and coefficient with 95% Confidence Interval for adjusted analysis.

### 3.3 Nontargeted proteomics and metabolomics analysis

#### 3.3.1 Baseline characteristics of the study subjects

The study included 5 male and 5 female PV patients aged 25–56 years (mean 40.1 ± 10.7 years), whose disease duration ranged from 252 days to 27 years (mean 11.2 ± 9.5 years), and their BMI ranged from 19.9 to 28.7 kg/m², with a mean of 25.0 ± 2.7 kg/m². The healthy control group comprised 5 males and 5 females aged 23–54 years, with a mean age of 38.8 ± 9.0 years and a mean BMI ranging from 17.8 to 25.7 kg/m² (21.9 ± 2.9 kg/m²). There were no statistically significant differences in age or sex between the two groups (P > 0.05). However, compared with the healthy controls, the PV patients had a significantly greater BMI (P = 0.005). (**Supplementary Table 7**).

#### 3.3.2 Nontargeted proteomic analysis

**Figure 3:**
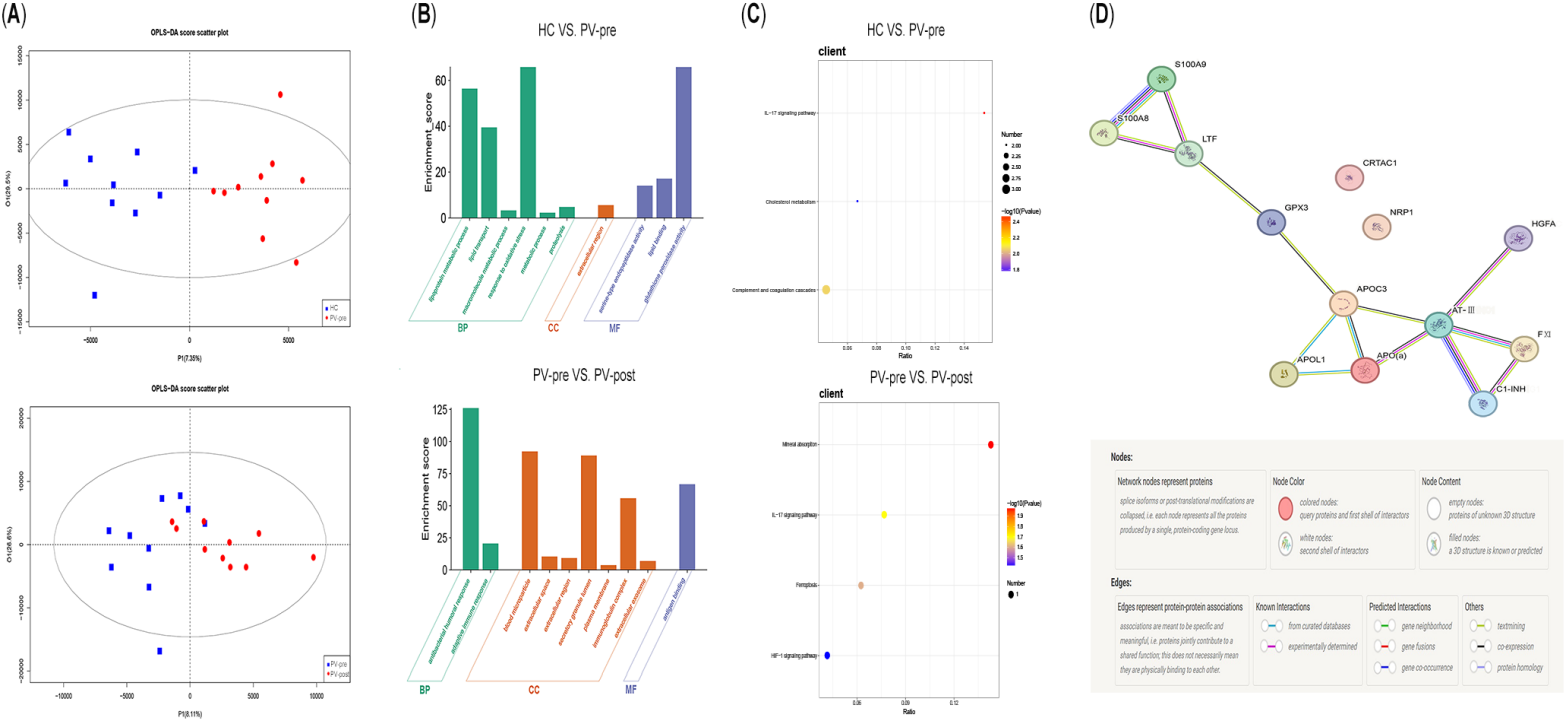
OPLS-DA model, proteomics map of differentially expressed protein enrichment pathways and protein interaction analysis of the genes in the STRING database. HCs: healthy controls; PV-pre: pre-IL-17Ai-treated psoriasis vulgaris group; PV-post: post-IL-17Ai treatment group. BP, biological process; CC, cellular component; GO, gene ontology; HCG, healthy control group; KEGG, Kyoto Encyclopedia of Genes and Genomes; MF, molecular function; OPLS-DA, orthogonal partial least squares discriminant analysis. **(A)** Scatter plot of OPLS-DA scores. P1 and Q1 denote the projected values under the first and orthogonal principal components, respectively. The OPLS-DA scores for the protein profiles of the HC vs. PV-pre and PV-pre vs. PV-post groups were significantly different. The scatter plot of the OPLS-DA scores shows significant separation between the HC vs. the PV-pre groups and the HC vs. the PV-post groups. **(B)** HC vs. PV-pre: GO enrichment histogram. A higher enrichment score on the vertical axis indicates that the target gene is more concentrated in the given function. The horizontal axis shows the GO names of the enriched proteins. Colors are used to differentiate between bars from different GO categories. A comparison of the GO enrichment analyses of the HCG and the PVG revealed significant enrichment at the BP level of lipoprotein metabolic process, lipid transport, macromolecule metabolic process, oxidative stress response, metabolic processes and proteolysis. At the CC level, the extracellular region was significantly enriched. At the MF level, lipoprotein binding, serine-type endopeptidase activity and glutathione peroxidase activity were significantly enriched. PV-pre vs. PV-post: enrichment at the BP level of the antibacterial humoral response and the adaptive immune response. At the CC level, blood microparticles, extracellular space, extracellular region, secretory granule lumen, plasma membranes, immunoglobulin complex and extracellular exosomes were enriched. At the MF level, antigen binding was enriched. **(C)** HC vs. PV-pre: Bubble plot of KEGG enrichment. The horizontal axis represents the ratio between the number of differentially expressed proteins and the background proteins in the given pathway. This reflects the degree of pathway enrichment under the experimental conditions. The dots on the vertical axis represent the different pathways. The dot size is representative of the number of differentially expressed proteins associated with the pathway, and the dot color is representative of the log10 value (p value). The darker the color is, the greater the statistical significance of the between-group difference in pathway enrichment; thus, the p value decreases. A comparison of the KEGG enrichment analyses of the HCG and the PVG revealed that the IL-17 signaling pathway, complement and coagulation cascades and cholesterol metabolism were enriched. PV-pre vs. PV-post: The IL-17 signaling pathway, ferroptosis pathway and mineral uptake pathway were significantly enriched. **(D)** Protein interaction analysis of the genes in the STRING database (https://cn.string-db.org/). nodes: represent proteins, with splicing subtypes and posttranslational modifications integrated and presented (i.e., all proteins produced by a single protein-coding gene locus are represented by the same node); node color: colored nodes represent target proteins and first-order interacting proteins, while white nodes represent second-order interacting proteins; node content: Hollow nodes represent proteins with unknown three-dimensional structures, while solid nodes represent proteins with well-defined three-dimensional structures; association edges: represent protein functional associations, which are specific and biologically meaningful (i.e., proteins that collaboratively participate in the same functional pathways, but physical binding is not necessarily required); interaction types include gene neighborhood, gene fusion, gene co-occurrence, text mining evidence, coexpression, and protein homology (**Supplementary Table 8**).

Nontargeted proteomics analysis of peripheral serum from the HC, PV-pre, and PV-post groups revealed a total of 1181 proteins. The OPLS-DA scatterplot revealed significant separation between the PV-pre/HC and PV-post/PV-pre OPLS-DA scores (**Figure 3**). This model confirmed that the differences between the two protein sample groups were reliable and significant.

Thirteen significantly altered differentially expressed proteins were identified in the PV-pre group compared with the HC group. Compared with those from healthy controls, peripheral blood from PV patients was upregulated in APO (a); APOC3, APOL1, S100A8, S100A9, and LTF were significantly upregulated; and AT-III, C1-INH, GPX3, HGFA, CRTAC1, NRP-1, and FXI were significantly downregulated (P < 0.05). Integrating the results of the GO and KEGG enrichment analyses revealed that the pathogenesis of PV involves primarily immune inflammation (S100A8/A9; KEGG: IL-17 signaling pathway), oxidative stress (GPX3; GO: oxidative stress response, glutathione peroxidase activity), lipid metabolism (APOC3, APO(a), APOL1; GO: lipoprotein metabolism, lipid transport, lipoprotein binding; KEGG: cholesterol metabolism) and complement-coagulation cascade (C1-INH, AT-III, FXI; GO: proteolysis, serine protease activity; KEGG: complement and coagulation cascade) dysregulation(**Table 2**; **Figures 3B**, **C**). Protein interaction analysis revealed that inflammation (S100A8, S100A9, LTF), oxidative stress (GPX3), lipid metabolism disorder (APOC3, APO(a), APOL1), and coagulation abnormalities (C1-INH, AT-III, FXI) form the core molecular mechanism chain of PV pathogenesis, with oxidative stress (GPX3) serving as a crucial link between inflammation and lipid metabolism (**Figure 3D**; **Supplementary Table 8**). Four differentially expressed proteins whose expression significantly changed between the PV-post and the PV-pre groups were identified. Following IL17Ai treatment, peripheral blood levels of immunoglobulin heavy constant gamma 1, immunoglobulin lambda variable 1-47, and serum transferrin increased, whereas those of S100A9 decreased, with statistically significant differences (P < 0.05). GO and KEGG enrichment analyses revealed that IL-17Ai primarily forms macromolecular immune complexes in the extracellular space, which are correlated with IL-17 signaling pathways and lipid peroxidation-induced ferroptosis processes (**Table 2**; **Figures 3B**, **C**; **Supplementary Materials-#8 Share Link;#10 Sheet6-13**).

**Table 2:** Differentially expressed proteins in peripheral blood samples from the HC, PV-pre and PV-post groups detected by untargeted proteomes.

Protein name	HC (n=10) vs. PV-pre (n=10)		FC	*P*-value	VIP
	HC (x̄ ± s)	PV-pre (x̄ ± s)			
APO(a)	333483.707 ± 202311.959	732538.577 ± 463227.427	2.197	0.014	3.104
APOC3	8267718.335 ± 2548322.832	12197153.300 ± 4558678.920	1.475	0.026	9.319
APOL1	309806.898 ± 78786.762	411068.855 ± 86811.744	1.327	0.014	1.033
S100A8	234871.643 ± 96751.662	406391.414 ± 139083.507	1.73	0.006	1.770
S100A9	306890.030 ± 121176.953	520463.183 ± 157710.160	1.696	0.003	2.107
LTF	128140.167 ± 35838.695	204707.098 ± 77828.145	1.598	0.011	1.168
AT-III	17380601.980 ± 2533358.387	14937635.700 ± 2632280.550	0.859	0.049	8.783
C1-INH	28241238.950 ± 5165750.799	23257027.700 ± 4638232.600	0.824	0.036	10.572
FXI	332973.510 ± 108696.952	238812.859 ± 42887.035	0.717	0.014	1.130
GPX3	2345529.607 ± 557600.739	1651287.010 ± 224114.016	0.704	0.002	4.141
HGFA	383807.679 ± 39626.502	285944.823 ± 54921.622	0.745	0.000	1.578
CRTAC1	212887.059 ± 64658.107	156573.157 ± 25837.506	0.735	0.014	1.116
NRP-1	304055.111 ± 52170.662	220229.200 ± 67108.955	0.724	0.006	1.166
Protein name	PV-pre (n=10) vs. PV-post (n=10)		FC	*P*-value	VIP
	PV-pre (x̄ ± s)	PV-post (x̄ ± s)			
IGLV1-47	40415.539 ± 19531.032	69670.040 ± 46011.679	1.724	0.038	1.056
IGHG1	3305068.220 ± 738215.825	4984904.830 ± 2751681.860	1.508	0.038	8.398
TF	2098069.570 ± 343921.351	2667856.860 ± 483062.823	1.272	0.008	3.700
S100A9	520463.183 ± 157710.160	367209.529 ± 151756.113	0.706	0.040	1.379

HCs, healthy controls; PV-pre, pre-IL-17Ai treatment group; PV-post, post-IL-17Ai treatment group. $\bar{x}$, mean; s, standard deviation; FC value is the ratio of PV-PG/HCG or PV-PTG/PV-PG; FC≥1.20 or ≤ 0.86, and P<0.05 indicates that the difference is statistically significant; VIP, variable importance projection. The test result unit is relative intensity. Differentially expressed molecules were screened based on uncorrected P-values (P < 0.05) and fold change (FC) thresholds of >1.5 or <0.67. Given the limited sample size, no statistically significant differentially expressed molecules were identified after FDR correction. These findings represent preliminary exploratory results.

#### 3.3.3 Lipid peroxidation pathway metabolomics study

Compared with those in healthy controls (HCs), 12 differential peripheral blood metabolites were detected in PV-pre patients. Compared with those in healthy individuals, peripheral blood levels of (+/-) 19 (20)-EpDPA were upregulated in PV patients, while those of PGF1a-1, PGF1a-2, PGB2-1, LTB5, PGD3, TXB3-1, RVE2-2, RVE2-3, RVD1-1, 17(R)-RVD1, and RVD1–3 were significantly downregulated (P < 0.05). Compared with those in the PV-pre group, seven differentially expressed metabolites were detected in the PV-post group. Following IL-17Ai treatment, peripheral blood levels of 20-HETE and RVD1–3 decreased significantly in PV patients, whereas the levels of the remaining lipid metabolites increased significantly (P < 0.05). The levels of four metabolites in the lipid peroxidation pathway (PGF1a-1, PGF1a-2, TXB3-1, and 17(R)-RVD1) were lower in the peripheral blood of PV patients than in that of healthy controls. Following IL-17Ai treatment, these metabolites were upregulated and improved. (**Table 3**; **Supplementary Materials-#10 Sheet 15-18**).

**Table 3:** Differentially abundant metabolites in peripheral blood samples from HCs, PV-pre patients, and PV-post patients detected by lipid oxidation pathway metabolomics.

Metabolite ID	HC (n=9*) vs. PV-pre (n=10)		FC	*P*-value
	HC (x̄ ± s)	PV-pre (x̄ ± s)		
PGF1a-1	32.940 ± 19.797	20.207 ± 8.863	0.613	0.009
PGF1a-2	81.638 ± 48.087	50.081 ± 23.271	0.613	0.049
PGB2-1	39.315 ± 18.371	27.933 ± 15.286	0.71	0.023
LTB5	64.06417.503	40.787 ± 22.6129	0.637	0.022
PGD3	7.929 ± 5.017	4.303 ± 3.093	0.543	0.018
TXB3-1	0.275 ± 0.188	0.191 ± 0.112	0.694	0.016
RVE2-2	0.584 ± 0.408	0.258 ± 0.238	0.442	0.002
RVE2-3	15.745 ± 8.032	9.901 ± 4.937	0.629	0.043
RVD1-1	1.604 ± 1.455	0.699 ± 0.685	0.436	0.03
17(R)-RVD1	0.482 ± 0.237	0.204 ± 0.158	0.424	0.011
RVD1-3	3.907 ± 2.126	2.487 ± 1.566	0.637	0.025
(+/-)19(20)-EpDPA	0.126 ± 0.136	0.210 ± 0.169	1.669	0.038
Metabolite ID	PV-pre (n=10) vs. PV-post (n=10)		FC	*P*-value
	PV-pre (x̄ ± s)	PV-post (x̄ ± s)		
PGF1a-1	20.207 ± 8.863	31.464 ± 16.237	1.557	0.042
PGF1a-2	50.081 ± 23.270	81.496 ± 43.423	1.627	0.036
20-HETE	23.628 ± 31.095	6.405 ± 7.298	0.271	0.028
TXB3-1	0.191 ± 0.112	0.450 ± 0.400	2.351	0.005
RVE1	0.177 ± 0.154	0.282 ± 0.162	1.593	0.014
RVD1-3	2.487 ± 1.566	1.553 ± 1.483	0.625	0.044
17(R)-RVD1	0.204 ± 0.158	0.337 ± 0.300	1.652	0.015

HCs, healthy controls; PV-pre, pre-IL-17Ai-treated psoriasis vulgaris group; PV-post, post-IL-17Ai treatment group. $\bar{x}$, mean; s: standard deviation; FC value is the ratio of PV-PG/HCG or PV-PTG/PV-PG; FC≥1.20 or ≤0.86, and P <0.05 indicates that the difference is statistically significant. *One patient in the healthy control group did not have a result because of insufficient sample volume. The test result unit is ng/ml. Differentially expressed molecules were screened based on uncorrected P-values (P < 0.05) and fold change (FC) thresholds of >1.5 or <0.67. Given the limited sample size, no statistically significant differentially expressed molecules were identified after FDR correction. These findings represent preliminary exploratory results.

## 4 Discussion

### 4.1 Ising network topology model guided by disease module theory: a critical tool for studying complex comorbidity networks in PV

Disease module theory posits that comorbidity in complex diseases arises from functionally connected nodes forming highly cohesive modules within biological networks, where intramodule connection density significantly exceeds intermodule connectivity. Core nodes serve as key drivers of module function, and their abnormalities can trigger functional disruption across the entire module. The cross-system characteristics of comorbidities stem from connections between modules via hub nodes, which act as “bridges”, facilitating interactions between different modules. The network of complex diseases thus forms a hierarchical structure composed of multiple modules interconnected through hub nodes (6). Using the Ising network topology model, we identified 11 comorbidity clusters centered on atherosclerosis-coronary heart disease (**Figure 2B**). These clusters represent functionally connected “disease modules” within the psoriasis systemic inflammatory network rather than random cooccurrences. The Ising model precisely captures module cohesion by quantifying internode dependencies. Degree centrality and expected influence centrality analyses of atherosclerosis and coronary heart disease confirm their status as “core nodes” in the PV comorbidity network, which is consistent with prior research highlighting the close association between PV and cardiovascular diseases (20). In contrast, traditional frequency analysis ranked atherosclerosis and coronary heart disease only 14th and 15th in terms of occurrence frequency, respectively. The Ising model’s centrality-based identification highlights its advantage in capturing module cores, demonstrating that core nodes are not necessarily high-frequency nodes but are functionally driven nodes. Atherosclerosis and coronary heart disease, as the “inflammatory core module”, drive the occurrence of other comorbidities within the module. Traditional PV comorbidity studies, which predominantly rely on incidence frequency and univariate associations, fail to identify and quantify module cohesion and the dynamic interactive effects among comorbidities (21).

In the Ising model, hub comorbidities with a median centrality >60 included pulmonary nodules, hypertension, fatty liver disease, osteoporosis, and spondylolisthesis. Among these, pulmonary nodules and hypertension exhibit the highest information transmission efficiency in the network (approaching centrality >0.0125). These comorbidities further interconnect with others to form a complex comorbidity network. These findings further support the perspective that “PV is not merely a dermatological condition but a systemic inflammatory disease” (2). The model revealed that pulmonary nodules connect to four comorbidity clusters simultaneously (emphysema-bullous emphysema-bronchiectasis, fatty liver-hyperlipidemia-type 2 diabetes, electrolyte imbalance-anemia, and thyroid dysfunction-benign tumors), positioning them as multisystem hub nodes. The close association between hypertension and the metabolic comorbidity cluster (fatty liver-type 2 diabetes-hyperlipidemia-hyperuricemia) as well as the stroke-arterial plaque comorbidity cluster suggests that PV shares a common pathological foundation with metabolic and cardiovascular diseases (e.g., insulin resistance and chronic low-grade inflammation) (3). Fatty liver plays a pivotal role in the hypertension–diabetes–hyperlipidemia–hyperuricemia (metabolic syndrome) comorbidity cluster, suggesting its potential involvement in the intrinsic mechanisms linking metabolic disorders and hypertension. Significant correlations exist among musculoskeletal disorders centered on osteoporosis and spondylolisthesis, suggesting that beyond psoriatic arthritis, broader musculoskeletal pathology modules warrant further attention in psoriasis patients.

The Ising network topology model of PV comorbidities reveals distinct hierarchical associations (**Figure 2B**): The first layer comprises the inflammatory core module (atherosclerosis–coronary heart disease), which drives systemic inflammation; the second layer consists of pivotal modules (pulmonary nodules, hypertension, fatty liver, osteoporosis, and spondylolisthesis), which transmit inflammatory signals from the core module to disseminate across metabolic, respiratory, and musculoskeletal systems; and the third layer comprises peripheral modules (e.g., stroke–arterial plaque, gastritis–intestinal polyps–peptic ulcer, dental disease–periodontitis, rhinitis–sinusitis), reflecting the “distant effects” of inflammation. This hierarchical structure is clearly revealed through the topological parameters of the Ising model (centrality gradient of core nodes-hub nodes-peripheral nodes), strongly suggesting that psoriasis, as a systemic inflammatory disease, exhibits a modular hierarchical comorbidity network rather than random multisystemic damage.

### 4.2 Clinical and multiomics studies provide preliminary evidence for disease module theory: associated comorbidity modules identified by Ising models share core molecular mechanisms

Pre-treatment multi-omics results for IL-17Ai showed that peripheral blood S100A8/A9 levels are elevated in psoriasis patients. As proinflammatory factors, they play a key role in initiating and amplifying the inflammatory response in psoriasis (22), while also serving as early warning indicators for cardiovascular events. APO(a) serves as the core structural protein of lipoprotein(a) [Lp(a)], Lp(a) has been confirmed by multiple studies as an independent predictor of coronary artery disease severity and future cardiovascular events (e.g., myocardial infarction, stroke). As the primary carrier of oxidized phospholipids (OxPL)—potent pro-inflammatory and pro-atherosclerotic mediators—APO(a) facilitates the transport of these harmful molecules. Therefore, APO(a) upregulation can be regarded as a key molecular link connecting the inflammatory state of psoriasis with atherosclerotic cardiovascular comorbidity (23). APOC3 is a key regulator that inhibits lipoprotein lipase and delays the clearance of triglyceride-rich lipoproteins. Elevated APOC3 levels lead to hypertriglyceridemia, and persistent hypertriglyceridemia constitutes a critical systemic metabolic environment for the development of fatty liver disease. It is also closely associated with insulin resistance and type 2 diabetes. Thus, APOC3 upregulation provides an upstream, shared molecular pathological basis for metabolic co-morbidity modules such as “fatty liver-type 2 diabetes-hyperlipidemia” within the Ising network (24). Meanwhile, downregulation of AT-III, C1-INH, and FXI—proteins involved in the complement-coagulation cascade (25) may represent the pathophysiological foundation for stroke co-morbidity in PV patients. GPX3, a crucial extracellular antioxidant enzyme, exhibits downregulated expression that may indicate insufficient systemic or local vascular oxidative stress defense capacity. Oxidative stress serves as a common substrate for psoriatic skin inflammation, atherosclerosis, and various metabolic disorders. Therefore, GPX3 abnormalities may serve as a common pathway biomarker linking chronic psoriatic inflammation to cardiovascular and metabolic comorbidities, explaining why these diseases tend to cluster in networks (26). Further GO and KEGG enrichment analyses confirmed activation of the IL-17 signaling pathway, oxidative stress, lipid metabolism disorders, and abnormalities in the complement-coagulation cascade in PV patients. Protein interaction analysis suggested that the core shared molecular mechanism chain underlying PV and its comorbidities involves the immune inflammation–oxidative stress–lipid metabolism disorder–coagulation abnormality pathway, with oxidative stress (GPX3) serving as a pivotal hub (**Figure 3D**). Moreover, Simultaneously downregulated lipid peroxidation metabolites include those with early compensatory anti-inflammatory effects (PGF1α-1, PGF1α-2, PGB2-1, LTB5, PGD3, TXB3-1) (27–30) and those with active anti-inflammatory effects in the mid-to-late stages of inflammation (RVE2-2, RVE2-3, RVD1-1, 17(R)-RVD1, RVD1-3 (31, 32). This finding indicates a defect in the anti-inflammatory mechanism of the lipid peroxidation pathway in PV. Multi-omics results following IL-17Ai treatment revealed downregulation of the proinflammatory factor S100A9 in peripheral blood of PV patients. Within the lipid peroxidation pathway, anti-inflammatory metabolites PGF1a-1, PGF1a-2, and TXB3-1, and 17(R)-RVD1 in the lipid peroxidation pathway. This anti-inflammatory effect of IL-17Ai was primarily mediated by blocking the IL-17 pathway and lipid peroxidation-induced ferroptosis (**Figures 3B**, **C**).

Clinical observations indicate that blocking the IL-17 signaling pathway with IL-17Ai leads to clearance of skin lesions in PV patients, along with significant improvement in early atherosclerosis ultrasound parameters (CCA-IMT). These findings further validate that IL-17-mediated immune inflammation drives PV pathogenesis. This pathway can trigger and promote PV and its core comorbidities through the “immunoinflammation–oxidative stress–lipid metabolism–coagulation abnormality” cascade while also driving the occurrence of other related comorbidities within the comorbidity network. Given the limited sample size and short follow-up period of this study, whether the observed early vascular improvements translate into reduced cardiovascular events requires confirmation through large-scale prospective studies.

### 4.3 Ising network topology model and multi-omics integration perspective: strategies and significance for clinical translation

#### 4.3.1 Guidelines for clinical screening and decision-making

**Figure 4:**
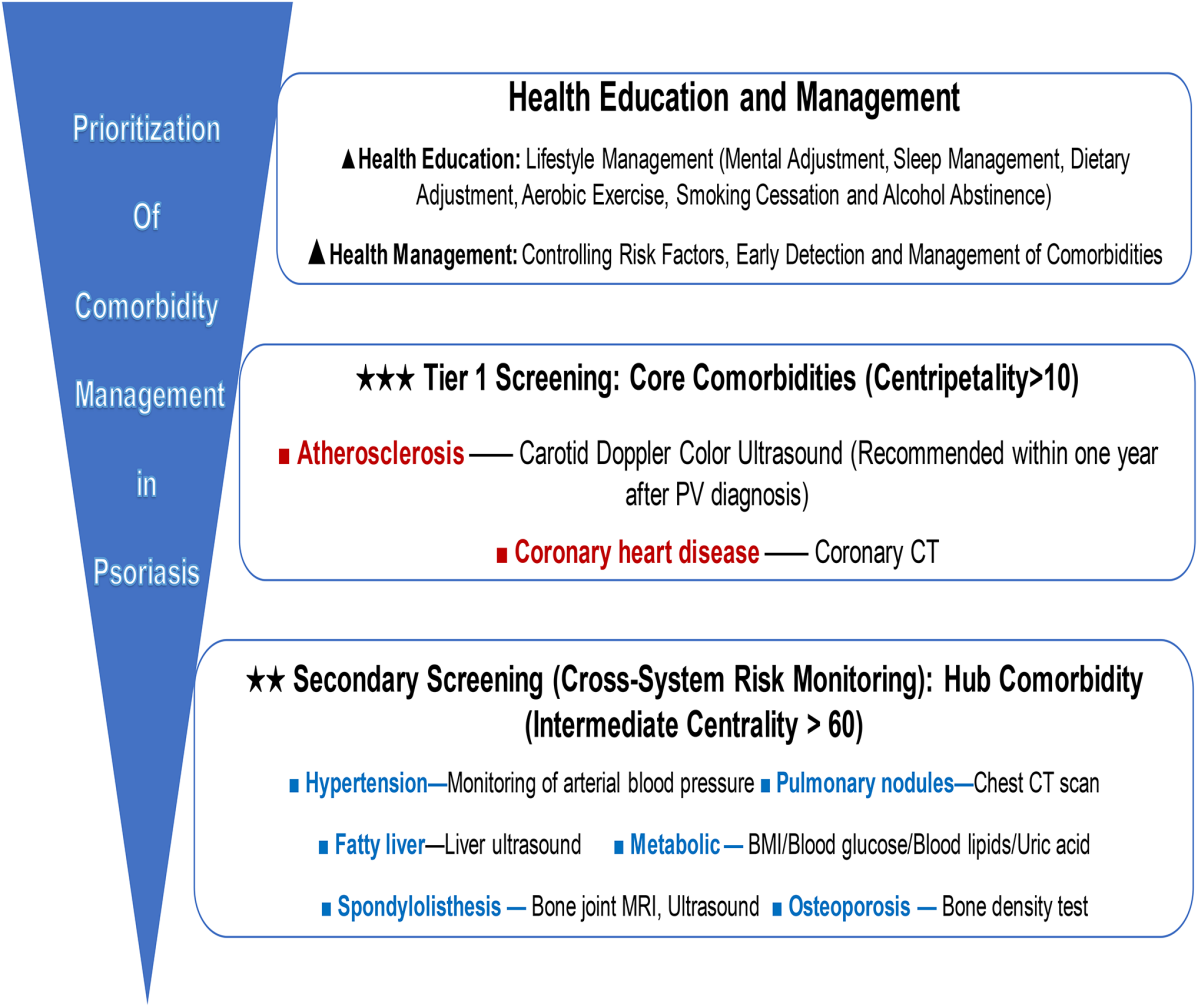
Prioritization of comorbidity management in psoriasis.

Within the complex PV comorbidity network, the core comorbidities and pivotal comorbidities identified via the Ising model should serve as priorities for clinical screening and monitoring, establishing a “comorbidity management priority” (**Figure 4**). Early identification and prevention/treatment of these key comorbidities can aid in the development of a model for clinical practice.

#### 4.3.2 Potential biomarkers

Differentially expressed proteins identified in proteomics studies hold promise as biomarkers for PV activity assessment, comorbidity risk screening, and treatment response evaluation. S100A8/A9 serves as an early risk indicator for cardiovascular events (33); although not PV specific, this inflammation-related factor can be used to assess PV inflammation severity and disease activity. Previous studies have indicated that hepatocyte growth factor activator (HGFA) regulates both hepatic lipid metabolism and vascular inflammation via the HGF/c-Met pathway (34), whereas chondrocalcinogen (CRTAC1) is crucial for maintaining joint cartilage integrity and function (35). Proteomics revealed downregulated HGFA expression in the peripheral blood of PV patients, elucidating its molecular basis as a cross-module hub in fatty liver disease. Downregulated CRTAC1 expression may underpin the pathophysiology of osteoarthritis comorbidity in PV patients, validating the theoretical prediction of “shared chondrodegenerative mechanisms within modules.” The value of CRTAC1 and HGFA as biomarkers for PV-associated osteoarthritis and fatty liver disease requires validation in larger cohorts.

#### 4.3.3 Therapeutic targets

The IL-17 signaling pathway, oxidative stress-related molecules, and lipid peroxidation metabolites identified in this study may serve as common therapeutic targets for PV and its key comorbidities. Our clinical study demonstrated that IL-17Ai has favorable efficacy and safety in treating PV, suggesting that blocking the IL-17 signaling pathway not only alleviates PV symptoms but also significantly improves early atherosclerosis (downregulation of CCA-IMT; P < 0.05). However, whether this ultimately reduces cardiovascular event risk requires validation through large-scale, long-term follow-up studies. Previous studies have indicated that GPX3 deficiency leads to mitochondrial dysfunction and increased lipid peroxidation, thereby exacerbating lipid metabolism disorders. As a link connecting immune inflammation, lipid metabolism disorders, and anticoagulation impairment, GPX3 holds promise as a core therapeutic target for preventing and treating inflammatory diseases, metabolic disorders, and cardiovascular diseases (26). Research has indicated that resolvins—anti-inflammatory lipid peroxidation metabolites—can resolve pulmonary inflammatory lesions, including pulmonary nodules (36). Resolvins, as anti-inflammatory, immunomodulatory, and antioxidant chemotherapeutic agents, have entered preclinical research (37). Our metabolomics findings revealed that anti-inflammatory mediators among lipid peroxidation metabolites in peripheral blood are significantly downregulated in PV patients. Blocking the IL-17 inflammatory pathway with IL-17Ai partially reversed this trend, leading to upregulation and improvement in PGF1a-1, PGF1a-2, TXB3-1, and 17(R)-RVD1 levels. These findings suggest that the proinflammatory mediator 17(R)-RVD1 may serve as a therapeutic target for PV and its inflammatory comorbidities. These findings provide theoretical and clinical evidence for the development of multitarget therapeutic strategies through network pharmacology to improve skin and systemic outcomes in PV patients.

### 4.4 Limitations and future directions

This study has several limitations: (1) Although the sample size is sufficiently large, the comorbidity Ising network topology model is based on data from a single-center hospital information system (HIS), and the multiomics analysis sample size is relatively small. This may introduce regional bias and instability in the omics results. Future multicenter, large-sample, multi-regional and multi-ethnic studies should be conducted for validation. (2) The specific functions of differentially expressed proteins and metabolites in PV and comorbidities require validation through subsequent *in vitro* functional experiments. (3) The cross-sectional study design makes it difficult to establish causal relationships between molecular factors and comorbidities, necessitating longitudinal studies. Future research should focus on (1) validating the potential of S100A8/A9, CRTAC1, HGFA, and other markers as risk indicators for comorbidities in longitudinal cohorts; (2) Given that GPX3 may serve as a pivotal hub in the core shared molecular mechanism chain underlying PV and its comorbidities, future mechanistic studies (such as cellular assays) will be conducted to validate GPX3’s function and explore its therapeutic potential. (3) performing mechanistic studies to elucidate the role of the IL-17 pathway in cross-system interactions (e.g., liver-bone-vascular); and (4) exploring network pharmacology-based “one-drug, multiple-target” strategies, such as developing novel therapies that simultaneously modulate IL-17 signaling and lipid peroxidation metabolism.

## Data Availability

The datasets presented in this study can be found in online repositories. The names of the repository/repositories and accession number(s) can be found in the article/**Supplementary Material**.

## Glossary

ANA: Antinuclear Antibody
IGRAs: Interferon-Gamma Release Assays
APO(a): Apolipoprotein(a)
IL17: Interleukin-17
APOC3: Apolipoprotein C3
IL17Ai: Interleukin-17A inhibitor
APOL1: Apolipoprotein L1
LTB5: Leukotriene B5
AS: Atherosclerosis
LTF: Lactotransferrin
AT-III: Antithrombin-III
MHz: Megahertz
BMI: Body mass index
MF: Molecular function
BP: Biological process
NRP1: Neuropilin-1
BSA: Body surface area
OPLS-DA: Orthogonal partial least squares discriminant analysis
CAD: Coronary Artery Disease
PASI: Psoriasis Area and Severity Index
CC: Cellular component
PGB2: Prostaglandin B2
CCA-IMT: Common Carotid Artery Intima-Media Thickness
PGD3: Prostaglandin D3
C1-INH: Plasma protease C1 inhibitor
PGF1α-1: Prostaglandin F1alpha-1
CRTAC1: Cartilage acidic protein1
PGF1α-2: Prostaglandin F1alpha-2
CT: Computed Tomography
PSV: Peak Systolic Velocity
DEP: differentially expressed protein
PV: Psoriasis vulgaris
DLQI: Dermatology life quality index
PV-pre: Psoriasis vulgaris pre-IL-17Ai treatment group
(+/-)19(20)-EpDPA: (+/-)-19(20)-Epoxy-4Z,7Z,10Z,13Z,16Z-docosapentaenoic acid
PV-post: Psoriasis vulgaris post-IL-17Ai treatment group
FC: Fold chang
RVD1-1: Resolvin D1-1
FXI: Coagulation factor XI
RVD1-3: Resolvin D1-3
GO: Gene ontology
17(R)-RVD1: 17(R)-Resolvin D1
GPX: Glutathione peroxidase
RVE2-2: Resolvin E2-2
HC: Healthy controls
RVE2-3: Resolvin E2-3
HGFA: Hepatocyte growth factor activator
S100A8: Protein S100-A8
HGF/c-Met: Hepatocyte Growth Factor/cellular Mesenchymal-epithelial transition factor
S100A9: Protein S100-A9
KEGG: Kyoto encyclopedia of genes and genomes
TXB3-1: Thromboxane B3-1
VIP: variable importance projection
ICA-LD: Lipid Droplets in Islet Cells.
