# Supplementary material for: Integrating clinical and multiomics evidence based on disease module theory: deciphering the comorbidity network of psoriasis vulgaris via the Ising model for mechanistic insights
Source: Front Immunol. 2026 Apr 14;17:1744789. doi: 10.3389/fimmu.2026.1744789 (PMC13121148; doi:10.3389/fimmu.2026.1744789)
Supplement: Supplementary file 1 [file DataSheet1.docx]

#11 Supplementary Methods

- 1. Proteomic experimental methods

3.1.1 High-abundance protein removal

Conventional high-abundance protein removal was first performed (15), followed by high-abundance protein depletion of the NP using the High Select Top14 Abundant Protein Depletion Mini SpinColumns kit (Thermo Fisher Scientific, USA) according to the manufacturer's instructions.

3.1.2 Protein quantification

First, BSA standard protein solutions and sample solutions were prepared using a Bradford protein quantification kit (Biyun Tian, China) according to the instructions. Triplicate replicates were performed for each concentration gradient. G250 staining solution was added, the absorbance was measured at 595 nm, and the protein concentration was calculated. Subsequently, 12% SDS‒PAGE gel electrophoresis, staining, and destaining were performed until the bands were clearly visible.

3.1.3 Liquid chromatography (LC) calibration

An iRT kit (Biognosys, Switzerland) and a calibrated LC system—a Vanquish™ Neo ultrahigh-performance liquid chromatograph (Thermo Fisher Scientific, USA)—were used.

3.1.4 Protein digestion

Enzymatic digestion, washing, and lyophilization were performed using DB protein digestion buffer and trypsin.

3.1.5 LC‒MS analysis in DIA

First, mobile phases A and B were prepared, and then the lyophilized powder was subjected to LC‒MS analysis. Raw mass spectrometry data were subsequently generated using a Vanquish Neo upgraded ultrahigh-performance liquid chromatography system coupled with a Thermo Fisher Scientific Orbitrap Astral mass spectrometer (Thermo Fisher Scientific, USA) and an Easy-spray (ESI) ion source.

3.1.6 Protein identification and quantification

For Blood-Plus (whole platform), raw file search analysis was performed using DIA-NN software (16) for deconvolution and species library searching.

3.1.7 Protein screening

Proteins with more than 40% inconsistent repeats or low abundance/missing values were screened out.

- 1. Experimental metabolomics methods

3.2.1 Lipid medium (LM) enrichment

Serum pretreatment followed previously reported methods (17), with key steps as follows: BHT, glycerol, and internal standards (ISs) were added to the serum. Lipid media were enriched using solid-phase extraction (SPE) columns. The eluates were dried via a vacuum concentrator. The dried samples were resuspended in 50 μL of acetonitrile/methanol (50/50, v/v) prior to LC‒MS analysis.

3.2.2 Instrumentation and experimental conditions

Lipid matrix analysis employed an LC‒MS system comprising a SCIEX 5500plus mass spectrometer (equipped with an ESI source; MA, USA) and a Thermo Fisher Scientific Dionex Ultimate 3000 high-performance liquid chromatograph (MA, USA). LC‒MS analysis was performed with a Waters ACQUITY UPLC BEH C18 column (2.1 × 50 mm, 1.7 μm). Mobile phase A was 0.1% acetic acid in water, and mobile phase B was acetonitrile/isopropanol (9/1, v/v). The gradient elution program is shown in Table 1. The flow rate was 0.4 mL/min, the column temperature was set at 40°C, the injection volume was 5 μL, and the data were acquired in negative ion and multiple reaction monitoring (MRM) mode. The gradient elution program for lipid medium analysis was as follows: time 0→1→8→10→10.01→12 min; A concentration 75→75→5→5→75→75%; B concentration 25→25→95→95→25→25% (eTable 4).

Supplementary Materials -#12 Supplementary Methods

Benjamini-Hochberg Method (BH Method)：

1. Sort all test p-values (p-values from t-tests) in ascending order；

2. For the i-th p-value, calculate the adjusted FDR value: FDR = (p-value × total number of tests) / rank i ;

3. Retain all results with FDR ≤ preset threshold (e.g., 0.05) as “significant differences”.

FDR < 0.05 serves as the criterion for determining significant differences.
