# Supplementary material for: Integrating clinical and multiomics evidence based on disease module theory: deciphering the comorbidity network of psoriasis vulgaris via the Ising model for mechanistic insights
Source: Front Immunol. 2026 Apr 14;17:1744789. doi: 10.3389/fimmu.2026.1744789 (PMC13121148; doi:10.3389/fimmu.2026.1744789)
Supplement: Supplementary file 2 [file Table1.docx]

**eTable1**

| **The top ten places of origin and the top six ethnic groups in the research cohort** | | | |
| --- | --- | --- | --- |
| **place of origin** | **Number of patients** | **Ethnicity** | **Number of patients** |
| **n (%)** | **n (%)** |  | **n (%)** |
| Hebei Province | 1264（26.41%） | Han Chinese | 5172 （94.40%） |
| Beijing Municipality | 542（11.32%） | Manchu | 119 （2.17%） |
| Shanxi Province | 404（8.44%） | Mongol | 80 （1.46%） |
| Inner Mongolia Autonomous Region | 359（7.50%） | Hui | 67 （1.22%） |
| Henan Province | 309（6.46%） | Korean | 8 （0.15%） |
| Heilongjiang Province | 279（5.83） | Tujia | 8 （0.15%） |
| Liaoning Province | 161（3.36%） |  |  |
| Jilin Province | 156（3.26%） |  |  |
| Anhui Province | 127（2.65%） |  |  |
| Shaanxi Province | 117（2.44%） |  |  |
| Total | 3718 (77.68) |  | 5454 （99.54%） |

4,786 patients included information on their place of origin. 5,479 patients included information on their ethnicity.

**eTable 2**

| **Comorbidity classification, names, ICD codes, and frequency of comorbidities in the psoriasis vulgaris cohort** | | | | | |
| --- | --- | --- | --- | --- | --- |
| Classification of Comorbidities | Diagnosis of the  78 Comorbidities | Detailed classification  of comorbidities | ICD-10  coding | Corresponding  codes for  comorbid names | Comorbidity frequency |
| Metabolic diseases | Non-insulin-dependent diabetes | Type 2 diabetes | E11.901 | S10 | 488 |
|  | Abnormal glucose tolerance |  | R73.0 | S11 | 32 |
|  | Gout NOS |  | M10.991 | S12 | 26 |
|  | Hyperlipidemia | Hyperlipoproteinemia, hypercholesterolemia | E78.501 | S13 | 1103 |
|  | Hyperuricemia |  | E79.001 | S14 | 695 |
| Cardiovascular diseases | Essential  hypertension^a^ | hypertension | I10 | S15 | 769 |
|  | Coronary atherosclerotic  heart disease | Myocardial infarction, angina,  coronary artery stenting  /bypass surgery | I25.1 | S16 | 132 |
|  | Congenital heart disease | Right heart congenital heart disease,  patent foramen ovale,  atrial septal defect,  patent ductus arteriosus,  tetralogy of Fallot | Q24. 901 | S17 | 3 |
|  | Valvular heart disease | After aortic valvuloplasty,  after heart valve replacement,  aortic valve calcification, aortic insufficiency,  mitral stenosis with aortic regurgitation,  mitral regurgitation,  mitral stenosis insufficiency,  mitral insufficiency,  tricuspid insufficiency,  tricuspid regurgitation | I38.X01 | S18 | 19 |
|  | Arrhythmia | Arrhythmias, sinus/atrial/ventricular tachycardia,  atrial premature contractions, sinus bradycardia,  sinus arrhythmias, atrial/ventricular/junctional/supraventricular premature contractions, atrial fibrillation, atrial flutter,  left/right bundle branch block, atrioventricular block,  ectopic rhythm, preexcitation syndrome,  postradio frequency ablation, palpitations,  ST changes, T wave changes | I49.9 | S19 | 413 |
|  | Hypertrophy of the  heart muscle | Dilated cardiomyopathy, hypertrophic cardiomyopathy,  left ventricular hypertrophy | I51.704 | S20 | 3 |
|  | Cardiac insufficiency | Cardiac function class I–IV | I50.90 | S21 | 6 |
|  | Arteriosclerosis |  | I70.903 | S22 | 1 |
|  | Atherosclerosis | Cerebral artery/carotid artery/coronary artery/aorta/lower  extremity atherosclerosis | I70.901 | S23 | 135 |
|  | Arterial plaque^b^ |  | ^--^ | S24 | 12 |
| Cerebrovascular diseases | Stroke | Cerebral hemorrhage, ischemic cerebrovascular disease,  cerebral infarction, cerebral infarction,  cerebral thrombosis | I64.X04 | S25 | 74 |
| Respiratory diseases | Emphysema NOS |  | J43.903 | S26 | 59 |
|  | Pulmonary nodules^b^ |  | ^--^ | S27 | 331 |
|  | Bullous emphysema | Pulmonary bulla | J43.901 | S28 | 50 |
|  | Pulmonary calcification |  | J98.404 | S29 | 12 |
|  | Chronic obstructive  pulmonary disease |  | J44.901 | S30 | 3 |
|  | Interstitial lung disease^c^ | Interstitial pneumonia, pulmonary interstitial fibrosis,  pulmonary fibrosis, pulmonary interstitial inflammation,  pulmonary interstitial lesions | ^--^ | S31 | 28 |
|  | Interstitial changes in the lung^b^ |  | ^--^ | S32 | 28 |
|  | Respiratory failure NOS |  | J96.901 | S33 | 0 |
|  | Bronchiectasis |  | J47.X01 | S34 | 8 |
| Liver and kidney diseases | Fatty liver^d^ | Alcoholic fatty liver, nonalcoholic fatty liver,  metabolic fatty liver | K76.001 | S35 | 1616 |
|  | Abnormal liver function | Liver damage, elevated aminotransferases | R94.501 | S36 | 625 |
|  | Cirrhosis | Hepatitis B cirrhosis, biliary cirrhosis,  drug-induced cirrhosis, alcoholic cirrhosis | K74.60 | S37 | 26 |
|  | Nephritis | Glomerulonephritis, occult nephritis,  nephritic syndrome, glomerulonephropathy,  IgA nephropathy, membranous nephropathy | N01-N03, N08 | S38 | 18 |
|  | Kidney stones |  | N20.0 | S39 | 113 |
|  | Hydronephrosis |  | N13.301 | S40 | 12 |
|  | Renal calcification | Calcification of renal papillary, intrarenal calcification,  renal calcification foci | N28.805 | S41 | 11 |
|  | Chronic kidney disease | Hypertensive nephropathy, diabetic nephropathy,  drug-induced nephropathy, nephrotic syndrome | N18.9 | S42 | 6 |
|  | Renal insufficiency^e^ | Renal impairment, abnormal renal function,  chronic kidney disease stage II–IV, renal failure | N19.X03 | S43 | 22 |
| Digestive system diseases | Gastritis | Atrophic and nonatrophic gastritis, verrucous gastritis,  superficial gastritis, erosive gastritis, reflux gastritis,  portal hypertensive gastritis, chronic gastritis | K29.703 | S44 | 69 |
|  | Peptic ulcer | Esophageal ulcers, pyloric ulcers,  gastric ulcers, duodenal ulcers | K27.904 | S45 | 15 |
|  | Bowel disease | Proctitis, chronic colitis, ulcerative colitis,  appendicitis, Crohn's disease | K63.951 | S46 | 10 |
|  | Intestinal polyps | Duodenal polyps, cecal polyps, colon polyps,  rectal polyps, and intestinal polyps | K63.802 | S47 | 20 |
|  | Gallstones | Gallstones, cholecystolithiasis | K80.203 | S48 | 190 |
|  | Gallbladder polyps |  | K82.808 | S49 | 182 |
|  | Constipation |  | K59.001 | S50 | 29 |
| Locomotor system | Osteoarthritis | Nonrheumatic osteoarthritis,  rheumatic osteoarthritis | M15-19,  M47 | S51 | 22 |
|  | Degenerative  osteoarthropathy^c^ | Joint degeneration,  hyperostosis and degenerative osteoarthropathy | ^--^ | S52 | 60 |
|  | Ligangitis/tenosynovitis  /fasciitis/synovitis^c^ | Bursitis, tenosynovitis, fasciitis, synovitis,  old knee ligament injury, neck ligament hypertrophy,  calcified/adhesive tendonitis, tendonopathy | ^--^ | S53 | 23 |
|  | Cervical spondylosis | Vertebral artery cervical spondylosis, radiculopathy, spondyloarthrosis with cervical spondylosis | M47.025  /121/225 | S54 | 31 |
|  | Vertebral spondylolisthesis | Intervertebral disc herniation/prolapse/bulge,  intervertebral disc/vertebral bone surgery | M51.805 | S55 | 73 |
|  | Necrosis of the femoral head | Avascular femoral head, aseptic necrosis of the femoral head, hip/femoral head replacement | M87.051  /951 | S56 | 20 |
|  | Old meniscus injury |  | M23.291 | S57 | 12 |
|  | Joint effusion |  | M25.491 | S58 | 12 |
|  | Osteoporosis NOS |  | M81.991 | S59 | 17 |
| Ocular system | Retinopathy |  | H35.006 | S60 | 9 |
|  | Cataract |  | H26.252 | S61 | 24 |
|  | Vitreous disorders | Vitreous opacity, after vitrectomy | H43.951 | S62 | 4 |
|  | Glaucoma NOS |  | H40.901 | S63 | 1 |
| Thyroid disease | Thyroid dysfunction | Primary/secondary hypothyroidism,  primary/secondary hyperthyroidism | R94.652 | S64 | 33 |
|  | Goiter | Nodular goiter, goiter | E04.901 | S65 | 10 |
| Nasal diseases | Snoring |  | R06.501 | S66 | 19 |
|  | Rhinitis |  | J31.001 | S67 | 25 |
|  | Sinusitis | Ethmoid sinusitis, maxillary sinusitis,  paranasal sinusitis, purulent sinusitis | J32.451 | S68 | 14 |
| Immune system | Autoimmune diseases | Systemic lupus erythematosus, systemic scleroderma,  pemphigus, bullous pemphigoid,  connective tissue disease | M35.903 | S69 | 38 |
| Dermatosis | Eczema |  | L30.902 | S70 | 68 |
|  | Dermatitis | Atopic dermatitis, seborrheic dermatitis, contact dermatitis,  allergic dermatitis, photosensitive dermatitis,  autosensitive dermatitis | L30.901 | S71 | 60 |
|  | Vitiligo |  | L80.X01 | S72 | 7 |
|  | Acne NOS |  | L70.901 | S73 | 46 |
|  | Scar |  | L90.502 | S74 | 10 |
|  | Superficial skin ulcer |  | L98.452 | S75 | 15 |
| Oral diseases | Dental disease^c^ | Root end cyst, pericoronitis, pulpitis purulent,  periapical abscess, septic gingivitis,  alveolar abscess, tooth defect, impacted tooth,  residual tooth root, residual crown | ^--^ | S76 | 39 |
|  | Periodontitis | Periodontal abscess, periodontal disease | K05.301 | S77 | 14 |
| Diseases of the Hematopoietic system | Anemia NOS | Mild/moderate/severe anemia, iron deficiency anemia,  hemorrhagic anemia, megaloblastic anemia,  thalassemia, renal anemia,  myelosuppressive anemia | D64.903 | S78 | 109 |
|  | Thrombocytopenia |  | D69.602 | S79 | 15 |
|  | Leukopenia |  | D70.X01 | S80 | 2 |
| Malnutrition | Hypoproteinemia |  | E77.801 | S81 | 47 |
| Electrolyte imbalances | Electrolyte imbalances | Hypokalemia, hypocalcemia, hypochloremia,  hypomagnesaemia, hypophosphatemia | E87.802 | S82 | 97 |
| Mental disorders | Mental disorders NOS | Depression, depressive state, anxiety disorder,  anxiety state, anxiety depression, bipolar disorder,  persistent mood disorder, schizophrenia | F06.952 | S83 | 38 |
| Infectious diseases | Infectious  diseases^c^ | EB virus infection, soft tissue infection of the skin,  oral fungal infection, HIV infection, sepsis cystitis,  balanitis, nasopharyngitis, tonsillar hypertrophy,  tonsillitis, hepatitis C, acute enteritis, shingles,  oral infection, urinary tract infection,  skin fungal infection, surgical wound infection,  soft tissue infection, upper respiratory tract infection,  helicobacter pylori infection,  novel coronavirus infection,  helicobacter pylori infection, vertebral infection,  hepatitis B, pneumonia, tuberculosis, bacteremia,  sepsis, herpes, pelvic inflammatory disease,  chronic bronchitis, syphilis, bronchitis, mumps,  otitis externa, cryptococcosis, otitis media, warts | ^--^ | S84 | 1149 |
| Tumor | Malignancy^c^ | Nasopharynx, thyroid, lung, mediastinum, esophagus,  stomach, liver, gallbladder, pancreas, kidney, bladder,  ureter, breast, endometrium, cervix, ovary,  prostate, skin, ileum, jejunum, colon,  rectum and bone malignancy;  secondary malignancies of lymph nodes and bone;  leukemia; elevated tumor markers | ^--^ | S85 | 55 |
|  | Benign  tumur^c^ | Hamartoma, pituitary prolactinoma, hepatic hemangioma,  benign skin tumors, breast hyperplasia,  breast nodules, benign uterine tumors,  mediastinal benign tumors, bone benign tumors,  gastrointestinal tract benign tumors | ^--^ | S86 | 298 |
|  | Cyst^c^ | Face/neck sebaceous glands cysts,  sinuses/nasopharynx/posterior auricular cysts,  epiglottis/thyroid/pineal gland/neuraxial canal/liver/kidney/spleen/pancreas/prostate/  ovaries/adnexa/epididymis and testicular cysts | ^--^ | S87 | 524 |

**^a^** Hypertension defined by the European Society of Cardiology (ESC) 2023 guidelines (office BP≥140/90 mmHg + ambulatory monitoring). **^b^** Arterial plaques belong to the category of ultrasound imaging, whereas interstitial changes in the lung and pulmonary nodules belong to the category of CT imaging; thus, they are not disease diagnoses and do not have corresponding ICD-10 codes. Nephritis includes acute nephritis N01 and chronic nephritis N03. **^c^** Interstitial lung disease, degenerative osteoarthropathy, ligangitis/tenosynovitis/fasciitis/synovitis, dental disease, infectious disease, malignancy, benign tumor and cyst; a total of 8 types of diseases have no corresponding ICD-10 codes. **^d^** Fatty liver as a diagnostic ultrasonography. **^e^** Stage II–IV chronic kidney disease classified as renal insufficiency.

**eTable 3**

| **Carotid artery Doppler ultrasound examination parameters, clinical significance, normal value, measurement position and phase** | | | | |
| --- | --- | --- | --- | --- |
| Parameter | Clinical Significance | Normal Range | Measurement Site | Measurement Timing |
| CCA-IMT | Assess early atherosclerosis | < 0.9 mm | Above the CCA, select a site 1.0-1.5 cm proximal to the bifurcation (bulb), typically measuring the posterior wall (distal wall); Avoid plaque locations if present. | Systolic phase |
| ICA-LD | Assess carotid stenosis | 5.0-6.0mm | 1.0–1.5 cm distal to ICA origin (carotid sinus). | Systolic phase |
| PSV | Assess carotid stenosis severity | CCA: 50-100cm/s; ICA: 50-90100cm/s; ECA: 60-110100cm/s | Identify and record the maximum PSV across multiple segments: CCA, carotid bulb, ICA, and ECA. | Highest flow velocity measured during systole. |

CCA-IMT: common carotid artery intima–media thickness; ICA-LD: internal carotid artery lumen diameter; PSV: peak systolic velocity; CCA: common carotid artery; ICA: internal carotid artery; ECA: external carotid artery.

**eTable 4**

| **Gradient elution program for LM analysis** | | |
| --- | --- | --- |
| Time/min | A% | B% |
| 0 | 75 | 25 |
| 1 | 75 | 25 |
| 8 | 5 | 95 |
| 10 | 5 | 95 |
| 10.01 | 75 | 25 |
| 12 | 75 | 25 |

**eTable 5**

| **Sample sizes of two subsets, baseline characteristics, and the proportion of top 10 comorbidity** | | | | | | | | | | | | | | |
| --- | --- | --- | --- | --- | --- | --- | --- | --- | --- | --- | --- | --- | --- | --- |
| **Group** | **Patient**  **Number** | **Sex**  **(male) n(%)** | **Age**  **n(±SD)** | **Comor**  **bidities** | **Type 2 diabetes (S10)** | **Hyper**  **lipidemia (S13)** | **Hyper**  **uricemia (S14)** | **Hyper**  **tension (S15)** | **Arrhythmia (S19)** | **Pulmonary nodules (S27)** | **Fatty liver (S35)** | **Abnormal liver function (S36)** | **Infectious diseases (S84)** | **Cyst (S87)** |
| **1993-2014** | 2608  (47.60%) | 1922 (73.70%) | 37.14  (±15.84) | 3302 | 163 (4.94%) | 356 (10.78%) | 156 (4.72%) | 251 (7.60%) | 117 (3.54%) | 1 (0.03%） | 666 (20.17%) | 198 (6.0%) | 457 (13.84%) | 173 (5.24%) |
| **2015-2024** | 2871 (52.40%) | 2068  (72.03%) | 39.68  (±16.73) | 7132 | 325 (4.56%) | 747 (10.47%) | 539 (7.56%) | 518 (7.26%) | 296 (4.15%) | 330 (4.63%） | 950 (13.32%) | 427 (5.99%) | 692  (9.70%) | 351 (4.92%) |
| **Statistical**  **analysis** | *P* = 0.0004  φ = 0.048 | *P* = 0.122 | *P* < 0.001  Cohen's = 0.156 |  | *P* = 0.320 | *P* = 0.548 | *P* < 0.001  φ= 0.048 | *P* = 0.477 | *P* = 0.107 | *P* < 0.001  φ=0.145 | *P* < 0.001  φ= 0.094 | *P*= 0.984 | *P* < 0.001  φ=0.060 | *P* = 0.434 |

Continuous variables (e.g., age): Student's t-test was used to assess mean differences between groups, with effect sizes reported using Cohen's d (<0.2 indicates a small effect). Binary variables (e.g., gender, smoking status): The chi-square test (χ²) was used to compare proportions between groups, with the Phi coefficient (φ) calculated as the effect size (<0.1 indicates a small effect). Comparison of two sample proportions: The two-sample proportion test (Z-test) was used. Categorical variables with small expected frequencies (e.g., lung nodule incidence): Fisher's exact test. Theoretical distribution fitting (e.g., distribution of case numbers): Chi-square goodness-of-fit test. All analyses were two-tailed, with P < 0.05 considered statistically significant.

**eTable 6**

| **Comparison of the efficacy of IL-17Ai therapy before and after treatment in PV patients** | | | |
| --- | --- | --- | --- |
|  | PV-pre (n=21) | PV-post (n=21) | *P*值 |
|  | *M (P25, P75)* | *M (P25, P75)* |  |
| DLQI | 8.0 (4, 12.5) | 0.0 (0.0, 0.0) | ＜0.001 |
| BSA | 12.0 (9.5, 17.5) | 0.0 (0.0, 1.0) | ＜0.001 |
| PASI | 14.4 (11.3, 18.2) | 0.0 (0.0, 1.3) | ＜0.001 |

PV, psoriasis vulgaris. PV-pre: Pre-IL-17Ai treatment group; PV-post: Post-IL-17Ai treatment group. DLQI: Dermatology Life Quality Index; BSA: Body Surface Area; PASI: Psoriasis Area and Severity Index. The values of the DLQI, BSA and PASI are nonnormally distributed and are represented by the median m and quartiles P25 and p75. The rank sum test and Friedman test of paired samples are used.

**eTable 7**

| **Basic information of the research subjects in the multiomics analysis** | | | | | | | | | |
| --- | --- | --- | --- | --- | --- | --- | --- | --- | --- |
| Number | Groups | Age | Sex | BMI | Duration (years) | DLQI | BSA | PASI | Comorbidity |
|  |  | (y) |  |  |  |  |  |  |  |
| 1 | HC | 54 | 0 | 18.3 | - | - | - | - | - |
|  | PV-pre | 56 | 0 | 22.6 | 3 | 4 | 6 | 6.3 | Hypertension, Hyperlipidemia |
|  | PV-post | 56 | 0 |  |  |  |  |  |  |
| 2 | HC | 23 | 1 | 20.7 | - | - | - | - | - |
|  | PV-pre | 25 | 1 | 24.8 | 2 | 4 | 12 | 14.4 | NV |
|  | PV-post | 25 | 1 |  |  |  |  |  |  |
| 3 | Control | 33 | 1 | 25.8 | - | - | - | - | - |
|  | PV-pre | 34 | 1 | 28.7 | 5 | 8 | 12 | 23.2 | Thyroid nodule |
|  | PV-post | 34 | 1 |  |  |  |  |  |  |
| 4 | HC | 41 | 1 | 25.1 | - | - | - | - | - |
|  | PV-pre | 46 | 1 | 26.3 | 11 | 3 | 20 | 26.1 | Type 2 Diabetes, Hyperlipidemia |
|  | PV-post | 46 | 1 |  |  |  |  |  |  |
| 5 | HC | 53 | 0 | 17.8 | - | - | - | - | - |
|  | PV-pre | 49 | 0 | 19.9 | 21 | 8 | 12 | 15.2 | Rhinitis |
|  | PV-post | 49 | 0 |  |  |  |  |  |  |
| 6 | HC | 52 | 1 | 25.7 | - | - | - | - | - |
|  | PV-pre | 55 | 1 | 22.9 | 27 | 5 | 7 | 13.8 | Hypertension, CAD, Hyperlipidemia |
|  | PV-post | 55 | 1 |  |  |  |  |  |  |
| 7 | HC | 34 | 0 | 23 | - | - | - | - | - |
|  | PV-pre | 37 | 0 | 28.4 | 12 | 19 | 9 | 11.4 | Dermatitis |
|  | PV-post | 37 | 0 |  |  |  |  |  |  |
| 8 | HC | 31 | 0 | 19.6 | - | - | - | - | - |
|  | PV-pre | 31 | 0 | 26.4 | 0.7 | 7 | 12 | 16.8 | NV |
|  | PV-post | 31 | 0 |  |  |  |  |  |  |
| 9 | HC | 34 | 1 | 20.6 | - | - | - | - | - |
|  | PV-pre | 35 | 1 | 24.5 | 7 | 8 | 15 | 17.9 | Rhinitis |
|  | PV-post | 35 | 1 |  |  |  |  |  |  |
| 10 | HC | 33 | 0 | 22.9 | - | - | - | - | - |
|  | PV-pre | 33 | 0 | 26 | 23 | 9 | 12 | 12.6 | NV |
|  | PV-post | 33 | 0 |  |  |  |  |  |  |

In the sex column, 1 is male and 0 is female; CAD: coronary atherosclerotic heart disease; BMI: body mass index; DLQI: Dermatology Life Quality Index; BSA: body surface area; PASI: Psoriasis Area and Severity Index;

HC: healthy control group; PV-pre: plaque psoriasis before IL17Ai treatment group; PV-post: plaque psoriasis after IL17Ai treatment group. In the sex column, 1 is male and 0 is female.

**eTable 8**

| **Protein name correspondence chart between the STRING database and our study** | |  |
| --- | --- | --- |
| Protein name in our study | Protein name |  |
|  | in STRING database |  |
| APO(a) | LPA |  |
| APOC3 | APOC3 |  |
| APOL1 | APOL1 |  |
| S100A8 | S100A8 |  |
| S100A9 | S100A9 |  |
| LTF | LTF |  |
| AT-III | SERPINC1/Antithrombin-III |  |
| C1-INH | SRPING1/Plasma protease C1 inhibitor |  |
| GPX3 | GPX3 |  |
| HGFA | HGFAC |  |
| CRTAC1 | CRTAC1 |  |
| NRP-1 | NRP1 |  |
| FXI | F11 |  |

HGFAC: Hepatocyte growth factor activator short chain; it activates hepatocyte growth factor (HGF) by converting it from a single chain to a heterodimeric form; it belongs to the peptidase S1 family.

Plasma protease C1 inhibitor: Activation of the C1 complex is under the control of the C1- inhibitor. It forms a proteolytically inactive stoichiometric complex with the C1r or C1s proteases and may play a crucial role in regulation. Antithrombin-III: The most important serine protease inhibitor in plasma that regulates the blood coagulation cascade, AT-III, inhibits thrombin and matriptase-3/TMPRSS7, as well as factors IXa, Xa and XIa.
